# Estrogen receptor modulators genistein, daidzein and ERB-041 inhibit cell migration, invasion, proliferation and sphere formation via modulation of FAK and PI3K/AKT signaling in ovarian cancer

**DOI:** 10.1186/s12935-018-0559-2

**Published:** 2018-05-01

**Authors:** Karen K. L. Chan, Michelle K. Y. Siu, Yu-xin Jiang, Jing-jing Wang, Thomas H. Y. Leung, Hextan Y. S. Ngan

**Affiliations:** Department of Obstetrics and Gynaecology, Queen Mary Hospital, University of Hong Kong, 6/F Professorial Block, Pokfulam, Hong Kong, SAR China

**Keywords:** Genistein, Daidzein, ERB-041, Ovarian cancer

## Abstract

**Background:**

Ovarian cancer is the most lethal gynaecological malignancy. Chemotherapy is the main stay of treatment for metastatic disease, with modest response rates but significant side effects. Therefore, there is a need for alternative therapies that can control the disease while offering good quality of life. Ovarian cancer cells express both estrogen receptor subtypes (ERα and ERβ). There is growing evidence that ERβ is anti-oncogenic. Genistein and daidzein are phytoestrogens found in soybeans and they display higher affinity to bind ERβ. ERB-041 is a potent selective ERβ agonist. In this study, we aimed to investigate the effects of genistein, daidzein and ERB-041 on ovarian cancer.

**Methods:**

Ovarian cancer cell lines were treated with genistein, daidzein and ERB-041 in pharmacological doses. Cell migration, invasion, proliferation, cell cycle arrest, apoptosis and sphere formation were assessed by Transwell migration and invasion assays, XTT assay, focus formation, flow cytometry and sphere formation assay, respectively. Immunoblotting analysis was performed to determine the downstream signaling pathways.

**Results:**

We found that genistein, daidzein and ERB-041 significantly inhibited ovarian cancer cell migration, invasion, proliferation, as well as induced cell cycle arrest and apoptosis. Significantly inhibitory effect on the size and number of sphere formed in genistein, daidzein and ERB-041 treated cells was also demonstrated. Moreover, genistein, daidzein and ERB-041 treatment reduced p-FAK, p-PI3K, p-AKT, p-GSK3β, p21 or cyclin D1 expression in ovarian cancer cells.

**Conclusion:**

Genistein, daidzein and ERB-041 decreased ovarian cancer cell migration, invasion, proliferation and sphere formation, and induced cell cycle arrest and apoptosis with altered FAK and PI3K/AKT/GSK signaling and p21/cyclin D1 expression, suggesting their roles on ovarian cancer cell metastasis, tumorigenesis and stem-like properties and their potential as alternative therapies for ovarian cancer patients.

**Electronic supplementary material:**

The online version of this article (10.1186/s12935-018-0559-2) contains supplementary material, which is available to authorized users.

## Background

Ovarian cancer is a common cancer in women, leading to the highest mortality among gynecological malignancies in the world [1]. Most patients (~ 75%) are diagnosed late with metastases. This, together with high rates of recurrence, contribute to its overall poor survival. Cancer stem-like cells (CSCs) is a small subpopulation of tumor cells bearing stem-like properties and is responsible for cancer initiation, progression, metastasis and recurrence [2]. Therefore, investigating alternative therapy that can regulate metastasis and stem-like properties may help ovarian cancer patients against this aggressive disease.

Ovarian cancer is believed to be a hormone responsive tumor since about 60–100% of tumors express estrogen receptors (ERs) [3]. There are two ER subtypes (ERα and ERβ) which differ in ligand binding specificity and show opposing functions on cell growth in various cancer cells [4]. Decreased ERβ expression was found during tumor progression [5], suggesting that ERβ may bear a protective role opposite to the tumor-promoting role of ERα. Functionally, exogenous expression of ERβ in ovarian cancer cells inhibited cell proliferation and motility, and increased apoptosis [6, 7].

Soy isoflavones are non-steroidal compounds found in plants, with similar chemical structure to 17-β-estradiol [8, 9] and thus considered as phytoestrogens. They can mimic the binding of estrogens to ERs, exerting estrogenic effects on target organs [8, 9]. Both epidemiological and clinical studies have found the healthy benefits of isoflavones related to many chronic diseases, including cardiovascular disease, postmenopausal symptoms, diabetes and cancers [8, 9]. In particular, some isoflavones are believed to have anticancer effects in malignancies such as breast, prostate, liver, lung, colon and gastric cancers [10]. Genistein and daidzein are two major isoflavones, constituting 60 and 30% of total isoflavones respectively found in soybeans [9]. They have higher affinities for ERβ than ERα [11, 12]. Genistein has been reported to inhibit cell proliferation, induce apoptosis, regulate cell cycle, modulate antioxidant effect and impair angiogenesis in both hormone-related and -unrelated cancer cells, including ovarian cancer [13]. Daidzein has also been shown to inhibit cell proliferation, affect cell cycle and angiogenesis in different types of cancer cells [8], whereas studies on daidzein on ovarian cancer were scanty, and the underlying mechanisms were still poorly defined.

Since the ligand-binding domains of ER subtypes are different and can be targeted by selective ligands, thus besides phytoestrogens, selective estrogen receptor modulators (SERMs) are gaining attention as alternative therapies for cancers [14]. Recently, we found ERα antagonist (MPP) and ERβ agonist (DPN) significantly suppressed ovarian cancer cell growth in vitro and in vivo, suggesting that targeting ER subtypes may be applicable to ovarian cancer patients [15]. ERB-041 is a potent, selective ERβ agonist and has been tested in phase 2 clinical trial for treatment of rheumatoid arthritis [16]. ERB-041 has been found to suppress UVB-induced skin cancer in a mouse model [17] and inhibit invasion of triple-negative breast cancer cells in vitro [18]. A recent finding revealed inhibition of ovarian cancer cell proliferation by ERB-041 [19], but overall studies of ERB-041 on ovarian cancer were very limited.

In the present study, we investigated the effects and downstream signaling of genistein, daidzein and ERB-041 on ovarian cancer metastasis, proliferation, cell cycle regulation, apoptosis and sphere formation in vitro. Our results suggest that genistein, daidzein and ERB-041 may be possible hormonal treatment options for ovarian cancer patients.

## Methods

### Chemicals

Soy isoflavones 5,7-dihydroxy-3-(4-hydroxyphenyl)-4H-1-benzopyran-4-one (genistein, G6776), 7-hydroxy-3-(4-hydroxy-phenyl)-4H-1-benzo-pyran-4-one, (daidzein, D7802) and the selective estrogen receptor modulator (SERM) 2-(3-fluoro-4-hydroxyphenyl)-7-vinyl-1,3-benzoxazol-5-ol (ERB-041, PZ0183), were purchased from Sigma-Aldrich (St Louis, MO) and dissolved in dimethyl sulfoxide (DMSO).

### Cell culture

Ovarian cancer cell lines SKOV-3, A2780CP and OVCAR-3 were used in this study. Cells were cultured in Dulbecco’s modified Eagle’s medium (DMEM) (Invitrogen, Grand Island, NY) supplemented with 10% fetal bovine serum (FBS) (Invitrogen) and antibiotics (50 U/ml penicillin and 50 µg/ml streptomycin) (Invitrogen) in a humidified atmosphere containing 95% air and 5% CO_2_ at 37 °C [15, 20]. SKOV-3 and OVCAR-3 were purchased from American Type Culture Collection (ATCC; Manassas, VA). A2780CP was kindly provided by Prof. B. Tsang, Department of Obstetrics and Gynecology, University of Ottawa).

### Immunoblotting

Cells plated in 6-well plates were treated with 10 (low pharmacological) and 50 µM (high pharmacological) genistein and daidzein and 0.01, 0.1 and 10 µM ERB-041 in complete medium. Control cells were treated with an equal amount of DMSO. After 24 h of treatment, cells were washed with PBS and harvested with Mammalian Cell Lysis Reagent CelLytic™ M (Sigma, C2978) containing protease inhibitor cocktail (Sigma, P8340), phosphatase inhibitor cocktail 2 (Sigma, P5726) and phosphatase inhibitor cocktail 3 (Sigma, P0044). After 15 min of incubation on a shaker at 4 °C, cells were scraped and centrifuged. The protein-containing supernatant was collected. 30 µg protein lysate was separated by 7.5% SDS-PAGE and electroblotted to polyvinylidene difluoride membranes (Bio-Rad Laboratories, Hercules, CA). The membranes were blocked with 5% skim milk and then hybridized with primary antibodies: anti-ERα (Dako, CA, M3634), anti-ERβ (Santa Cruz, sc-8974), anti-p-FAK (Cell Signaling, Danvers, 8556), anti-FAK (Santa Cruz, sc-558), anti-p-PI3K85 (Abcam, ab138364), anti-p-AKT (Cell Signaling, 4060), anti-AKT (Cell Signaling, 4691), anti-p-GSK3β (Cell Signaling, 5558), anti-GSK3β (Cell Signaling, 12456), anti-p21 (Santa Cruz, sc-397), anti-cyclin D1 (Santa Cruz, sc-753), anti-E-cadherin (Cell Signaling, 3195), anti-Vimentin (Cell Signaling, 5741) and anti-Actin (Sigma, A5060) at 4 °C overnight. The membranes were then washed and probed with appropriate secondary antibodies conjugated with horseradish peroxidase (Santa Cruz) at room temperature for 1 h. After washing, the blots were detected with Clarity Western Enhanced Chemiluminescence Substrate (Bio-rad) and visualized by autoradiography [15, 20].

### In vitro migration and invasion assays

For genistein, daidzein and ERB-041 treatments, cells were treated with 10 and 50 µM genistein and daidzein, 0.01, 0.1 and 10 µM ERB-041 or DMSO. For transient knockdown of ERα and ERβ in SKOV-3, cells were transfected with siRNAs of ERα (Santa Cruz, Santa Cruz, CA, sc-29305) and ERβ (Santa Cruz, sc-35325) using SilentFect™ (Bio-Rad Laboratories, Hercules, CA) for 48 h before cell counting and cell plating. Control siRNA (sc-37007) was used as control. Cells (1.25 × 10^5^) were plated in DMEM medium on the upper compartment of a Transwell chamber. Then, cells were migrated through an 8-µm pore size membrane or invaded through a Matrigel-coated membrane (Corning^®^ BioCoat™ Matrigel^®^ Invasion Chambers) toward lower chamber with DMEM plus 10% FBS (as a chemoattractant). After 8–24 h, cells on the upper side of the membrane were removed with a cotton bud and the migrated or invaded cells were then fixed, stained, and counted under a light microscope [20, 21].

### Cell proliferation assay

Cell proliferation was determined by XTT assay and foci formation assay [15]. For XTT assay, 3000 cells per wells were seeded in 96-well plates overnight. Cells were washed with PBS and treated with 1, 5, 10 and 50 µM genistein and daidzein, 0.01, 0.1 and 10 µM ERB-041 or DMSO in complete medium. Cell proliferation was measured 24 and 48 h after treatment using Cell Proliferation Kit II (Roche Biosciences, Indianapolis, IN, USA) according to manufacturer’s instructions in an Infinite^®^ 200 microplate reader at 492 nm (Tecan Group Ltd, Männedorf, Switzerland).

For foci formation assay, A2780CP cells per wells were seeded in 6-well plates at a density of 500 cells per well overnight. Cells were washed with PBS and treated with 10 and 50 µM genistein and daidzein, 0.01, 0.1 and 10 µM ERB-041 or DMSO in complete medium. At day 7 after treatment, cells were fixed and stained with 1% crystal violet (Sigma-Alrich). Numbers of foci were counted.

### Cell cycle analysis

SKOV-3 cells were treated with 50 µM genistein and daidzein, 10 µM ERB-041 or DMSO for 48 h. Harvested cells were washed in PBS and fixed in cold 70% ethanol at 4 °C overnight. Cells were then washed, treated with 50 μl RNase (100 μg/ml, Sigma) and stained with 200 μl propidium iodide (50 μg/ml, Invitrogen) in dark for 20 min. Green fluorescent-stained cells were analyzed by flow cytometry using a FACSCanto flow cytometer (BD, San Jose, CA) and FlowJo software. Cell-cycle distribution was presented as percentage of the G1, S, and G2/M phases of cells.

### Apoptosis assay

SKOV-3 cells were treated with 50 µM genistein and daidzein, 10 µM ERB-041 or DMSO for 48 h. Adherent and floating cells were harvested, washed in PBS and stained with Annexin-V-FLUOS Staining Kit (Roche) according to manufacturer’s protocol. The double-stained cells were then analyzed by flow cytometry using a FACSCanto flow cytometer (BD).

### Sphere formation assay

OVCAR-3 sphere forming cells established by suspension culture in stem cell-condition medium (serum-free DMEM/F12 supplemented with 20 ng/ml human recombinant epidermal growth factor, 10 ng/ml human recombinant basic fibroblast growth factor and antibiotics penicillin and streptomycin) (Invitrogen) using ultra-low attachment 6-well plates (Corning) were treated with genistein (10 µM), daidzein (10 µM), ERB-041 (0.1 and 10 µM) or DMSO for 7 days. Spheres generated were photographed and sphere number were counted under light microscope [22].

### Statistical analysis

Statistical analysis was performed using the Prism Software Package (GraphPad Software, San Diego, CA). The results were expressed as the mean ± standard deviation. Two-tailed Student’s t test was used. *p *< 0.05 was considered statistically significant.

## Results

### ERα and ERβ expression in ovarian cancer cell lines

**Fig. 1:**
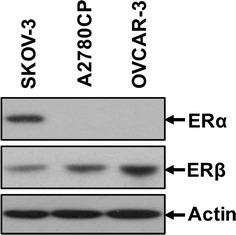
ERα and ERβ expression in three ovarian cancer cell lines, SKOV-3, A2780CP and OVCAR-3 as determined by immunoblotting

We first examined the expression of ERα and ERβ in three ovarian cancer cell lines, SKOV-3, OVCAR-3 and A2780CP by Western blot analysis (Fig. 1). ERα was expressed in SKOV-3, but not in OVCAR-3 and A2780CP, whereas ERβ was expressed in all three cell lines.

### Genistein, daidzein and ERB-041 inhibited ovarian cancer cell migration and invasion

**Fig. 2:**
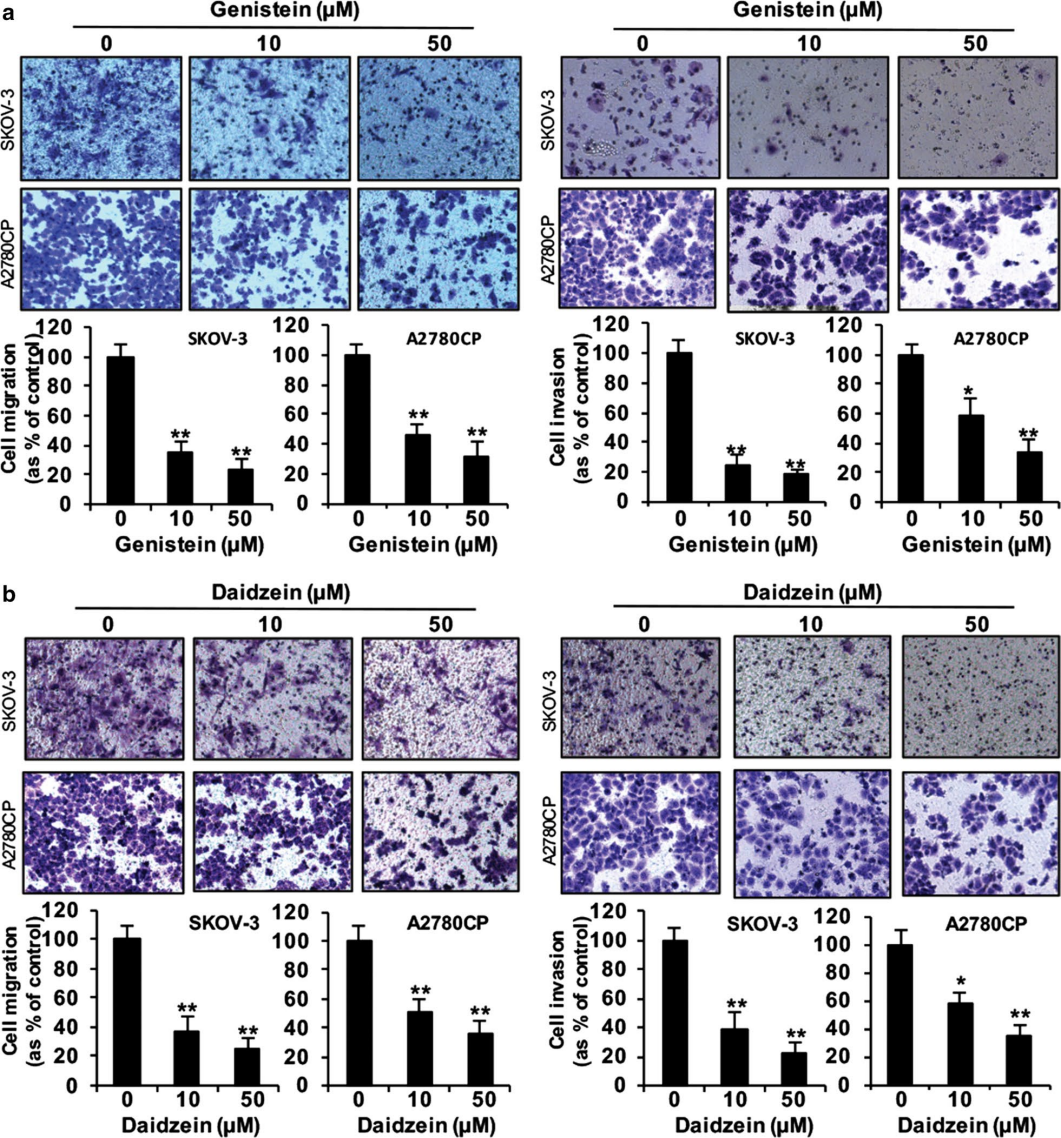
Genistein and daidzein inhibited ovarian cancer cell migration and invasion. In vitro migration and invasion assays in SKOV-3 and A2780CP cells treated with **a** genistein or **b** daidzein in low (10 µM) and high (50 µM) doses. Upper panel: representative images of migrating or invading cells. Lower panel: Cell migration or invasion presented as percentage of control; n = 3; *p < 0.05; **p < 0.005

Next, we determined the effect of genistein, daidzein and ERB-041 on ovarian cancer cell migration and invasion. Transwell migration and invasion assays revealed significantly reduced migration and invasion in genistein and daidzein treated cells compared with that in control cells dose-dependently in both SKOV-3 and A2780CP cells (all *p *< 0.05) (Fig. 2). Low (10 µM) and high (50 µM) doses of genistein inhibited cell migration by 65 and 77% in SKOV-3 cells and 54 and 68% in A2780CP cells, respectively (Fig. 2a, left panel), whereas 10 and 50 µM of daidzein inhibited cell migration by 63 and 75% in SKOV-3 cells and 49 and 64% in A2780CP cells, respectively (Fig. 2b, left panel). The inhibition on cell invasion was 76 and 82% in SKOV-3 cells and 41 and 66% in A2780CP cells by 10 and 50 µM genistein, respectively (Fig. 2a, right panel), whereas the inhibition was 62 and 78% in SKOV-3 cells and 43 and 66% in A2780CP cells by 10 and 50 µM daidzein, respectively (Fig. 2b, right panel).

**Fig. 3:**
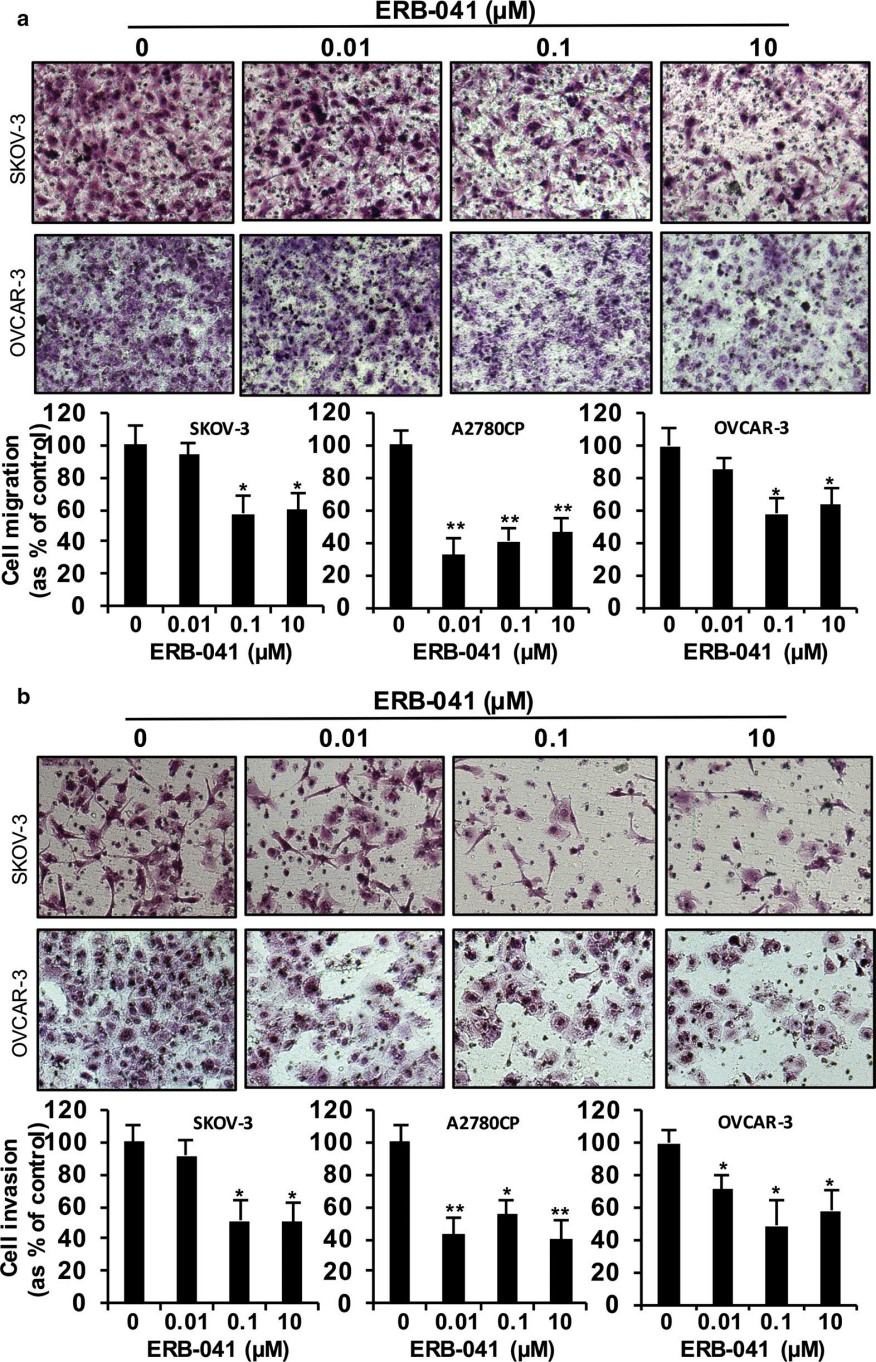
ERB-041 reduced ovarian cancer cell migration and invasion. In vitro **a** migration and **b** invasion assays in SKOV-3, A2780CP and OVCAR-3 cells treated with different doses of ERB-041 (0.01, 0.1 and 10 µM). Upper panel: representative images of migrating or invading cells. Lower panel: cell migration or invasion presented as percentage of control; n = 3; *p < 0.05; **p < 0.005

Significantly reduced cell migration and invasion in ERB-041 treated cells was also demonstrated by Transwell migration and invasion assays when compared with that in control cells in SKOV-3, A2780CP and OVCAR-3 cells (all *p *< 0.05) (Fig. 3). ERB-041 significantly reduced cell migration by 68% at the minimum dose of 0.01 µM in A2780CP cells, 43 and 42% at 0.1 µM in SKOV-3 and OVCAR-3 cells, respectively (Fig. 3a). 40, 54 and 37% inhibition on cell migration was detected in SKOV-3, A2780CP and OVCAR-3 cells by 10 µM ERB-041 treatment, respectively (Fig. 3a). At the minimum dose of 0.01 µM ERB-041 in A2780CP and OVCAR-3 cells, 57 and 29% reduction on cell invasion was demonstrated, respectively (Fig. 3b). 10 µM ERB-041 significantly reduced cell invasion by 50, 60 and 42% in SKOV-3, A2780CP and OVCAR-3 cells, respectively (Fig. 3b).

### Knockdown of ERβ increased ovarian cancer cell migration and invasion

**Fig. 4:**
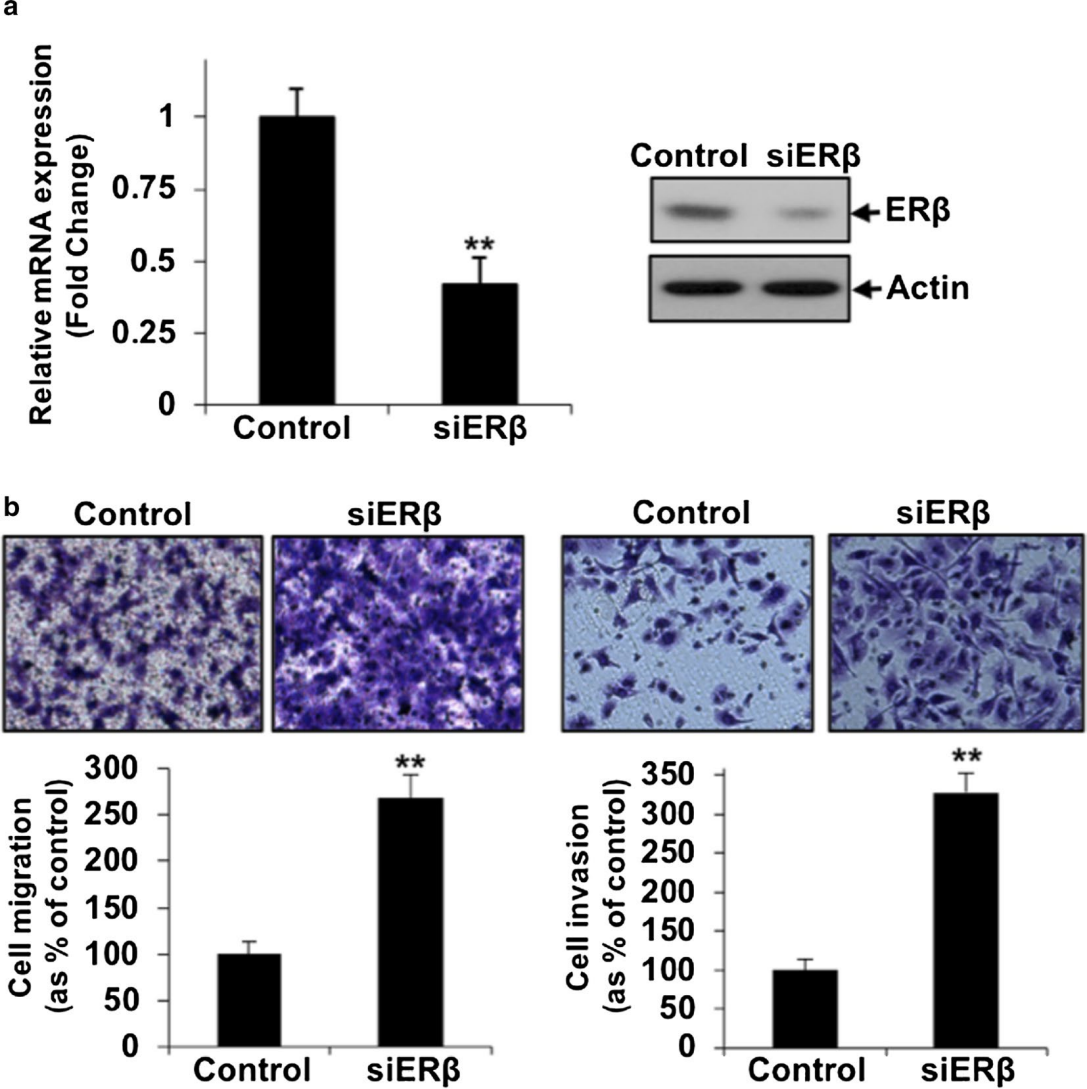
Transient silencing of ERβ induced ovarian cancer cell migration and invasion. **a** Transient knockdown of ERβ (siERβ) mRNA and protein expression in SKOV-3 cells by qPCR and immunoblot analysis respectively. **b** In vitro migration and invasion assays in SKOV-3 cells transient transfected with siRNA specifically targeting ERβ and control siRNA. Upper panel: representative images of migrating or invading cells. Lower panel: cell migration or invasion presented as percentage of control; n = 3; **p < 0.005

We showed the inhibitory effect of genistein, daidzein and ERB-041 on ovarian cancer cell migration and invasion. We next examined if knockdown of ERβ would display opposite effect. mRNA and protein expression of ERβ was evaluated by qPCR and immunoblotting at 48 and 72 h after siRNA transfection, respectively. siERβ profoundly reduced ERβ mRNA and protein expression in SKOV-3 cells relative to the non-target control siRNA cells (Fig. 4a). Silencing of ERβ significantly increased cell migration and cell invasion (Fig. 4b). We also performed transient knockdown of ERα in SKOV-3 cells and found that silencing of ERα had no significant change on cell migration and invasion (Additional file 1: Figure S1).

### Genistein, daidzein and ERB-041 reduced ovarian cancer cell proliferation

**Fig. 5:**
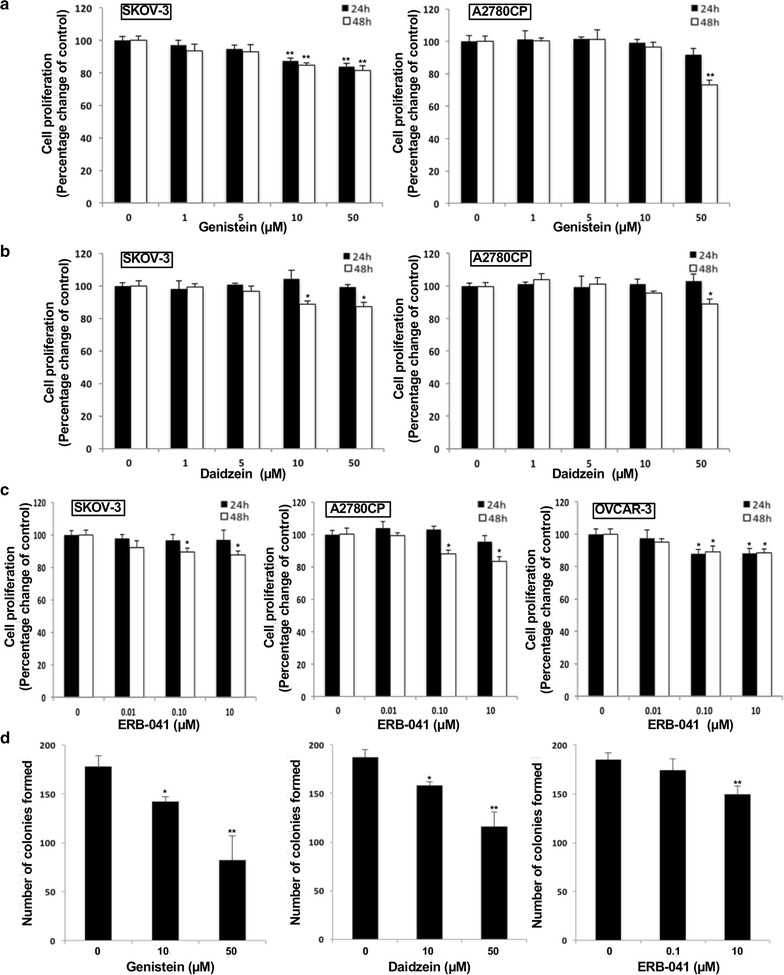
Genistein, daidzein and ERB-041 decreased ovarian cancer cell proliferation. XTT assays in SKOV-3, A2780CP or OVCAR-3 cells treated with different doses of **a** genistein (1, 5, 10 and 50 µM), **b** daidzein (1, 5, 10 and 50 µM) or **c** ERB-041 (0.01, 0.1 and 10 µM) for 24 and 48 h. Cell proliferation presented as percentage of control; n = 3; *p < 0.05; **p < 0.005. **d** Focus formation assay in A2780CP cells treated with different doses of genistein, daidzein (10 and 50 µM) or ERB-041 (0.1 and 10 µM) presented as number of colonies formed. n = 3; *p < 0.05; **p < 0.005, significantly different from the control

To determine if the inhibitory migration and invasion effect of genistein, daidzein and ERB-041 is due to reduced cell proliferation, XTT assay was performed 24 and 48 h after genistein, daidzein and ERB-041 treatment in SKOV-3, A2780CP or OVCAR-3 cells. We found that genistein, daidzein and ERB-041 inhibited cell proliferation in a dose- and time-dependent manner (Fig. 5a–c). Although we found 50 µM of genistein and daidzein and 0.1 and 10 µM ERB-041 showed significantly inhibitory effect on cell proliferation ranging from 11 to 26% in SKOV-3, A2780CP or OVCAR-3 cells (all *p *< 0.05), their effects on reduction of cell migration and invasion are much more prominent (Figs. 2, 3, 5).

Besides XTT assays, we applied focus formation assay to investigate the effect of genistein, daidzein and ERB-041 on ovarian cancer cell proliferation. We found the number of colonies from genistein and daidzein treated A2780CP cells decreased dose-dependently (Fig. 5d). 10 µM genistein and daidzein could cause significant inhibition by 20 and 16%, respectively (Fig. 5d). Genistein and daidzein at 50 µM caused 54 and 38% decrease on colonies formation, respectively (Fig. 5d). 0.1 µM ERB-041 did not have significant effect on focus formation, whereas 10 µM inhibited focus formation by 20% (Fig. 5d).

### Genistein, daidzein and ERB-041 induced ovarian cancer cell cycle arrest

**Fig. 6:**
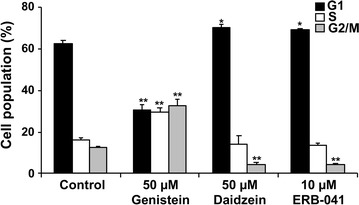
Genistein induced S and G2/M cell cycle arrest, whereas daidzein and ERB-041 promoted G1 cell cycle arrest. Cell cycle distribution of SKOV-3 cells treated with genistein (50 µM), daidzein (50 µM) or ERB-041 (10 µM) by flow cytometry presented as percentage of cell population in G1, S and G2/M phase of cell cycle of control. n = 3; *p < 0.05; **p < 0.005, significantly different from the control

We next investigated the effect of genistein, daidzein and ERB-041 on cell cycle progression in SKOV-3 cells by flow cytometry analysis using PI staining. 50 µM genistein induced S and G2/M cell cycle arrest, whereas 50 µM daidzein and 10 µM ERB-041 induced G1 cell cycle arrest (Fig. 6).

### Genistein, daidzein and ERB-041 promoted ovarian cancer apoptosis

**Fig. 7:**
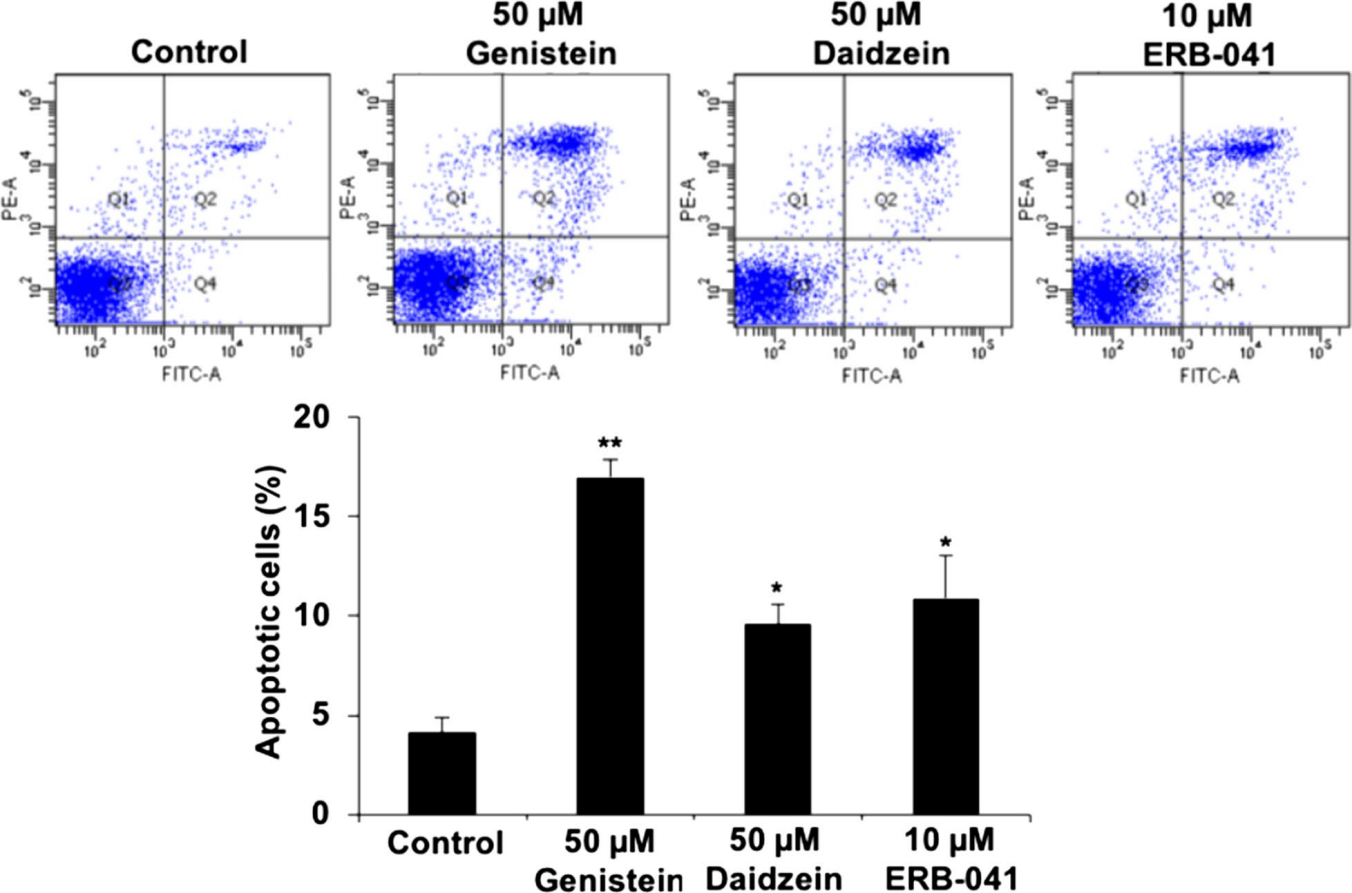
Genistein, daidzein and ERB-041 enhanced ovarian cancer apoptosis. The apoptosis status of SKOV-3 cells treated with genistein (50 µM), daidzein (50 µM) or ERB-041 (10 µM) by Annexin-V-FLUOS Staining presented as percentage of apoptosis. n = 3; *p < 0.05; **p < 0.005

The apoptosis effect of genistein, daidzein and ERB-041 in SKOV-3 cells was analyzed by flow cytometry analysis using Annexin V/PI double staining. The percentage of apoptotic cells was significantly increased in 50 µM genistein (17%), 50 µM daidzein (9.6%) and 10 µM ERB-041 (10.9%) when compared to control cells (4.2%) (Fig. 7).

### Genistein, daidzein and ERB-041 attenuated ovarian cancer sphere formation capacity

**Fig. 8:**
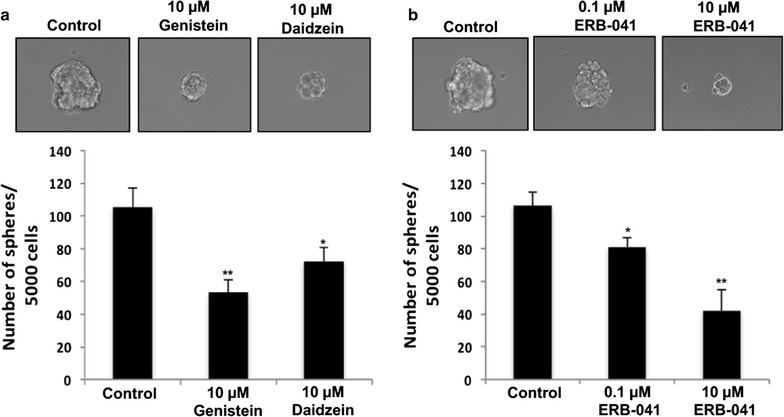
Genistein, daidzein and ERB-041 retarded ovarian cancer sphere formation capacity. Sphere formation assay in OVCAR-3 cells treated with **a** genistein (10 µM), daidzein (10 µM) or **b** ERB-041 (0.1 and 10 µM) for 7 days. Left panel: representative images of sphere forming cells showing the size of sphere formed. Right panel: sphere formation presented as number of spheres formed. n = 3; *p < 0.05; **p < 0.005

Our finding that genistein, daidzein and ERB-041 could reduce ovarian cancer migration and invasion prompted us to examine their roles on the regulation of stem-like properties because metastasis was one of the characteristics of CSCs. We then evaluated the capacity of OVCAR-3 cells treated with genistein (10 µM), daidzein (10 µM), ERB-041 (0.1 and 10 µM) or DMSO (vehicle) to grow as multi-cellular spheroids in stem-condition culture systems by sphere formation assay. Results showed that genistein and daidzein treated OVCAR3 cells formed smaller and less sphere cells (50 and 32% decrease, respectively) than that of control cells (all *p *< 0.05) (Fig. 8a). Dose dependent inhibitory effect was observed in ERB-041 treated cells (Fig. 8b). Smaller and less sphere cells (24 and 58% decrease, respectively) were formed in 0.1 and 10 µM treated OVCAR-3 cells compared with that of control cells (all *p *< 0.05) (Fig. 8b). The inhibition of ovarian cancer sphere formation suggested that genistein, daidzein and ERB-041 could regulate stem-like properties in ovarian cancer cells.

### Genistein, daidzein and ERB-041 inhibited activation of FAK and PI3K/AKT/GSK signaling and modulated p21 or cyclin D1 expression in ovarian cancer cells

**Fig. 9:**
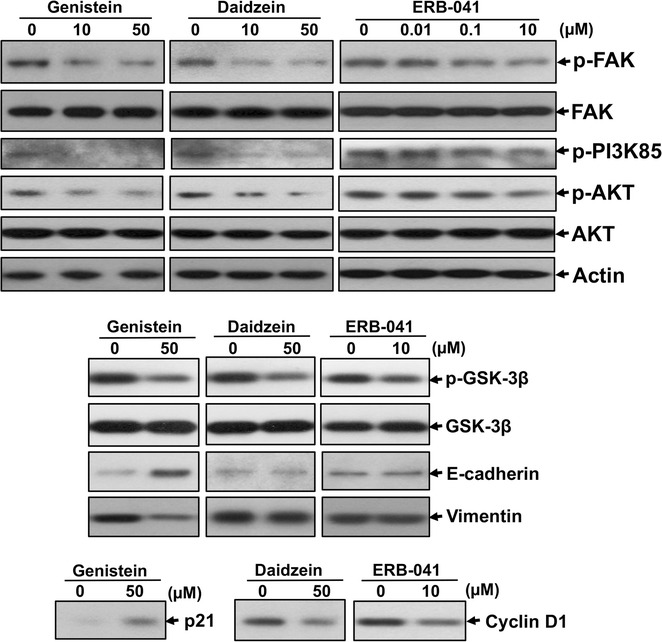
Genistein, daidzein and ERB-041 suppressed FAK and PI3K/AKT/GSK signaling and modulated expression of cell cycle regulators or EMT markers in ovarian cancer cells. Immunoblot analysis on p-FAK, FAK, p-PI3K85, p-AKT, AKT, p-GSK, GSK, p21, cyclin D1, E-cadherin or vimentin in SKOV-3 cells treated with different doses of genistein (10 or 50 µM), daidzein (10 or 50 µM), ERB-041 (0.01, 0.1 or 10 µM) or DMSO (vehicle)

Next, we investigated the possible downstream signaling by which genistein, daidzein and ERB-041 mediated their effect on ovarian cancer by immunoblotting. Focal adhesion kinase (FAK) is a non-receptor protein tyrosine kinase that has a critical role on cancer cell migration and invasion [23]. AKT is a serine/threonine kinase that plays an important role in many cellular processes, such as cancer cell proliferation, survival, migration and stem cell regulation [24]. We found that genistein, daidzein and ERB-041 treated SKOV-3 cells reduced FAK and AKT activation as detected by phosphorylation on Tyr^397^ and Ser^473^, respectively (Fig. 9). Moreover, genistein, daidzein and ERB-041 also suppressed phosphorylation of Phosphatidylinositol 3 kinase (PI3K)85 and GSK3β, upstream and downstream of AKT respectively [25, 26], suggesting that genistein, daidzein and ERB-041 could modulate PI3K85/AKT/GSK3β signaling in ovarian cancer (Fig. 9). We also examined expression of cell cycle regulators p21 and cyclin D1 [27, 28] as well as epithelial–mesenchymal transition (EMT) markers E-cadherin and Vimentin [29] in genistein, daidzein and ERB-041 treated cells. We detected increased p21 and E-cadherin, and reduced vimentin expression in genistein treated SKOV-3 cells (Fig. 9). Daidzein and ERB-041 treated cells decreased cyclin D1 expression, but have no virtual effect on E-cadherin and Vimentin expression (Fig. 9).

## Discussion

Hormonal therapy is an attractive treatment due to its relatively few side effects [30]. Ovarian cancer cells express both estrogen receptor subtypes (ERα and ERβ), which exert opposite effects on carcinogenesis [15]. Due to the anti-oncogenic role of ERβ, targeting ERβ in various cancers has received a considerable amount of attention in recent years [31]. Genistein and daidzein are two major isoflavones present in soybean products and also are commercially available. They are considered as phytoestrogens which can mimic the binding of estrogen to ERs with higher affinity for ERβ than ERα [11]. Such higher affinity to ERβ make them considered as SERMs [13]. Epidemiological studies found that soy isoflavone intake was associated with a reduced risk of ovarian, breast and prostate cancers in China and Japan [32]. Among soy isoflavones, genistein had been most widely investigated and was found to display anti-neoplastic activity in various cancers. Thus, genistein was believed to be a potential chemopreventive and chemotherapeutic agent [13]. In this study, we investigated the effects of genistein and daidzein on ovarian cancer cell migration, invasion, proliferation, cell cycle, apoptosis and sphere formation in pharmacological doses. Since it was postulated that the effects of genistein and daidzein could be mediated via ERβ activation, we also studied the effects of ERB-041, a highly selective synthetic ERβ agonist with 200-fold higher affinity for ERβ than ERα [33].

The anti-proliferative effect of genistein and daidzein has been studied in various types of cancer, including breast, prostate, colon, pancreatic and ovarian cancers [10, 34]. Genistein augmented ovarian cancer cell proliferation by inducing G2/M cell cycle arrest and altering cell cycle checkpoint pathway [13, 35]. Daidzein has also been shown to inhibit ovarian cancer cell proliferation [36]. A recent finding demonstrated the inhibitory effect on ovarian cell proliferation by ERB-041 [19]. Our current data showing inhibition of ovarian cancer cell proliferation by genistein, daidzein and ERB-041 is consistent with previous findings and supports a possible protective role of ERβ in ovarian cancer. In fact, increased cell proliferation was documented in ovarian cancer cells after ectopic expression of ERβ [6, 7], whereas opposite effect was demonstrated in ERβ-depleted ovarian cancer cells [19].

To further investigate if the anti-proliferative effect of genistein, daidzein and ERB-041 is mediated by altering cell cycle progression, cell cycle analysis was performed. Our findings revealed genistein promoted S and G2/M cell cycle arrest, whereas daidzein induced G1 cell cycle arrest. Genistein induced G2/M cell cycle arrest has been reported in various cancer cells, including ovarian cancer cells [6, 7, 13]. Modulation of S and G2/M phases by genistein has also been detected in head and neck squamous cell carcinoma [37] and neuroblastoma [38]. Daidzein induced cell cycle arrest in the G1 phase in ovarian cancer cells as presented in the current study suggested that genistein and daidzein induced cycle arrest via different pathways. Such observation has been demonstrated in melanoma cells [39], gastric [40] and prostate cancer cells [28]. The relationship between the stage-specific cell cycle arrest and structure of genistein and daidzein has been suggested [39]. Similar to daidzein, we further showed ERB-041 promoted G1 cell cycle arrest which concur with its effect on cell cycle regulation in skin cancer cells [17].

PI3K/AKT signaling, an important regulator of cellular functions including cell proliferation, has been found to be overexpressed and activated in ovarian cancers [26]. Cell proliferation is tightly linked to cell cycle progression. p21 is a cyclin-dependent kinase inhibitor which negatively regulate cell cycle progression such as G2/M cell cycle arrest [28]. Upregulation of p21 by inhibition of PI3K/AKT pathway has been reported. The inhibition of PI3K/Akt pathway by its specific inhibitor LY294002 is able to upregulate the expression of p21 [41]. Moreover, genistein-induced G2/M arrest has been found to be associated with upregulated p21 expression in breast, prostate and lung cancer [39]. Our data showed that genistein treatment suppressed PI3K/AKT phosphorylation which was accompanied with increased expression of p21. These results suggested that inactivation of PI3K/AKT signaling associated with induction of p21 is involved in genistein-induced G2/M arrest in ovarian cancer. Activated AKT could also inactivate GSK3β by phosphorylation of Ser9 [25], which in turn leading to stabilization of cyclin D1, an important regulator for G1/S phase transition [27]. Reduced expression of p-PI3K85, p-AKT and cyclin D1 by ERB-041 treatment has been reported in skin cancer cells [17]. We found that daidzein and ERB-041 decreased p-PI3K85, p-AKT, p-GSK3β, and cyclin D1 expression, suggesting PI3K/AKT/GSK3β/cyclin D1 signaling is linked to daidzein and ERB-041 induced G1 cycle arrest in ovarian cancer.

Genistein has been shown to induce apoptosis via suppression of AKT signaling in ovarian cancer [13]. The current data also displayed genistein induced apoptosis associated with AKT inactivation. Daidzein has also reported to induce apoptosis in breast [42] and hepatic [43] cancer cells. Erb-041-mediated apoptosis in UVB-induced skin tumors has been documented [17]. Our data demonstrated induced apoptosis by daidzein and Erb-041 treatment in ovarian cancer probably via inactivation of AKT signaling.

Apart from the anti-proliferative effects, genistein, daidzein and ERB-041 had also been found to impair cell migration and invasion in other cancers but these effects on ovarian cancers were much less reported. In breast cancer cells, genistein and daidzein diminished cell invasion by inhibiting NF-κB pathway [44]. Genistein could bind to and inhibit mitogen-activated protein kinase kinase 4 (MEK4) kinase activity in prostate cancer cells, which in turn attenuated matrix metalloproteinase-2 (MMP-2) expression and reduced cell invasion [45]. The inhibitory effect on cell invasion by genistein treatment in hepatocellular carcinoma has also been documented [46]. In a mouse model, ERB-041 has been found to suppress skin cancer invasiveness via PI3K-AKT pathway and WNT signaling [17]. A recent study also revealed ERB-041 decreased invasiveness of triple-negative breast cancer cells [18]. In this study, we found that pharmacological doses of genistein, daidzein and ERB-041 could also impair ovarian cancer cell migration and invasion. On the contrary, we observed increased cell migration and invasion in ERβ-depleted ovarian cancer cells, confirming the present ERβ agonists function on cell migration and invasion.

We also found that the inhibitory effects on cell migration and invasion mediated by genistein, daidzein and ERB-041 were associated with the suppression of FAK activation, indicating that FAK pathway might be involved. Overexpression and activation of FAK have been shown in numerous cancers and are associated with poor prognosis including in ovarian cancer [47]. In preclinical studies, small molecule FAK inhibitors retarded tumor growth and metastasis. A safe and well-tolerated FAK inhibitor has also been reported in a clinical trial study [47]. Besides FAK signaling, EMT also plays a critical role on cancer metastasis. Suppression of EMT by upregulating E-cadherin and downregulating vimentin by genistein in ovarian cancer has been demonstrated which was in agreement with our current data [29]. However, E-cadherin and vimentin expression remained unchanged in daidzein and ERB-041 treated ovarian cancer cells. Dose-dependent increase of E-cadherin in ERB-041 treatment in skin cancer has been documented with 20 µM as the lowest dose [17], our data showed 10 µM ERB-041 could not altere E-cadherin expression in ovarian cancer cells, suggesting higher dose may be needed which could be further studied in future study.

CSCs is a small subpopulation of tumor cells bearing stem-like properties and is responsible for cancer initiation, progression, metastasis and recurrence, thus considered as a potential therapeutic target in human cancers [2]. In breast cancer cells, genistein augmented CSC regulation via the Hedgehog pathway [48]. Genistein also inhibited gastric cancer stem-like properties [22]. A recent study has documented 7-Difluoromethoxyl-5,4′-di-n-octyl genistein, a synthetic genistein analogue, attenuated ovarian cancer stem-like properties by downregulating FOXM1 [49]. Our findings demonstrated altered sphere formation by genistein, daidzein and ERB-041 treatment, suggesting their role on the regulation of stem-like properties. Moreover, increasing evidence has linked AKT pathway to cancer stem cell regulation [24]. In the present study, since we also detected decreased AKT activation in genistein, daidzein and ERB-041 treated ovarian cancer cells, inhibition of AKT signaling might be related to genistein, daidzein and ERB-041 mediated retarded sphere formation.

## Conclusion

According to the NIH Clinical Trials database, Phase I/II trials of genistein in breast, prostate, pancreatic and metastatic colorectal cancers are ongoing or completed. Phase II trial of Erb-041 has also been conducted for its potential use in rheumatoid arthritis [16]. Moreover, selective ERβ agonist has been tested in phase 2 clinical trial for treatment of menopausal symptoms [50]. Besides anti-proliferation role of genistein and daidzein, the present data further demonstrated the inhibitory roles of genistein, daidzein and ERB-041 on ovarian cancer cell migration, invasion and sphere formation, indicating their roles on cell metastasis and regulation of stem-like properties. Our preclinical study revealed the anti-oncogenic roles of genistein, daidzein and ERB-041 in ovarian cancer and provided some insight into the mechanisms involved in the possible protective roles of soy products in ovarian cancers. Our findings support future studies to investigate their potential use in clinical settings as possible treatment options in ovarian cancer patients.

## Additional file

**Additional file 1: Figure S1.** Transient silencing of ERα did not show significant change in ovarian cancer cell migration and invasion. **a** Transient knockdown of ERα (siERα) mRNA expression in SKOV-3 cells by qPCR. **b** In vitro migration and invasion assays in SKOV-3 cells transient transfected with siRNA specifically targeting ERα and control siRNA. Upper panel: representative images of migrating or invading cells. Lower panel: cell migration or invasion presented as percentage of control; n = 3;**p < 0.005.

## Abbreviations

CSCs: cancer stem-like cells
DMEM: Dulbecco’s modified Eagle’s medium
DMSO: dimethyl sulfoxide
EMT: epithelial–mesenchymal transition
ERs: estrogen receptors
FAK: focal adhesion kinase
FBS: fetal bovine serum
MEK4: mitogen-activated protein kinase kinase 4
MMP-2: matrix metalloproteinase-2
PI3K: phosphatidylinositol 3 kinase
SERMs: selective estrogen receptor modulators
