# Supplementary material for: Estrogen receptor modulators genistein, daidzein and ERB-041 inhibit cell migration, invasion, proliferation and sphere formation via modulation of FAK and PI3K/AKT signaling in ovarian cancer
Source: Cancer Cell Int. 2018 May 1;18:65. doi: 10.1186/s12935-018-0559-2 (PMC5930957; doi:10.1186/s12935-018-0559-2)

Figure S1. Transient silencing of ERα did not show significant change in ovarian cancer cell migration and invasion. (A) Transient knockdown of ERα (siERα) mRNA expression in SKOV-3 cells by qPCR. (B) *In vitro* migration and invasion assays in SKOV-3 cells transient transfected with siRNA specifically targeting ERα and control siRNA. Upper panel: representative images of migrating or invading cells. Lower panel: cell migration or invasion presented as percentage of control; n=3; **, $p<0.005$.

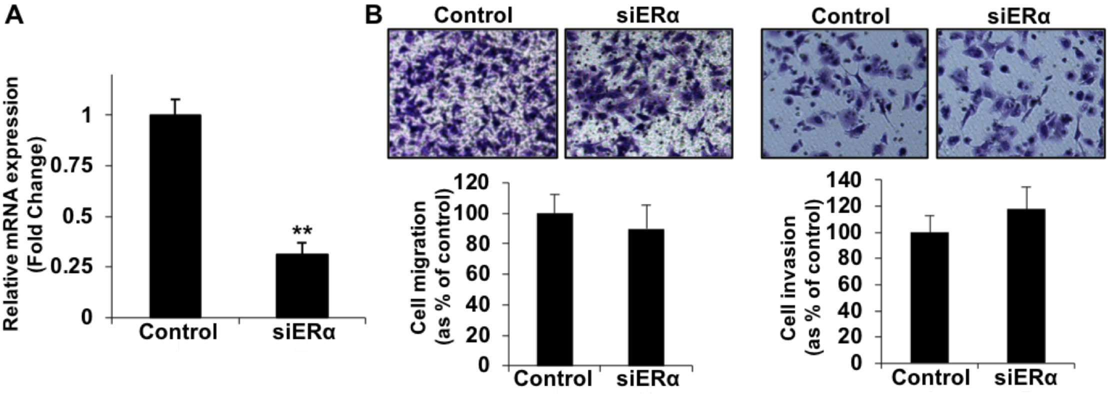

Supplement: Supplementary file 1 — Additional file 1: Figure S1. Transient silencing of ERα did not show significant change in ovarian cancer cell migration and invasion. a Transient knockdown of ERα (siERα) mRNA expression in SKOV-3 cells by qPCR. b In vitro migration and invasion assays in SKOV-3 cells transient transfected with siRNA specifically targeting ERα and control siRNA. Upper panel: representative images of migrating or invading cells. Lower panel: cell migration or invasion presented as percentage of control; n = 3;**p < 0.005. [file 12935_2018_559_MOESM1_ESM.pdf]
